# EP300 Protects from Light-Induced Retinopathy in Zebrafish

**DOI:** 10.3389/fphar.2016.00126

**Published:** 2016-05-19

**Authors:** Reiko Kawase, Yuhei Nishimura, Yoshifumi Ashikawa, Shota Sasagawa, Soichiro Murakami, Mizuki Yuge, Shiko Okabe, Koki Kawaguchi, Hiroshi Yamamoto, Kazumi Moriyuki, Shinsaku Yamane, Kazuhiro Tsuruma, Masamitsu Shimazawa, Hideaki Hara, Toshio Tanaka

**Affiliations:** ^1^Department of Molecular and Cellular Pharmacology, Pharmacogenomics, and Pharmacoinformatics, Mie University Graduate School of MedicineTsu, Japan; ^2^Mie University Medical Zebrafish Research CenterTsu, Japan; ^3^Department of Systems Pharmacology, Mie University Graduate School of MedicineTsu, Japan; ^4^Department of Omics Medicine, Mie University Industrial Technology Innovation InstituteTsu, Japan; ^5^Department of Bioinformatics, Mie University Life Science Research CenterTsu, Japan; ^6^Ono Pharmaceutical Co, Ltd.Osaka, Japan; ^7^Molecular Pharmacology, Department of Biofunctional Evaluation, Gifu Pharmaceutical UniversityGifu, Japan

**Keywords:** light-induced retinopathy, EP300, STAT3, apoptosis, comparative transcriptome analysis, zebrafish, systems pharmacology

## Abstract

Exposure of rhodopsin to bright white light can induce photoreceptor cell damage and degeneration. However, a comprehensive understanding of the mechanisms underlying light-induced retinopathy remains elusive. In this study, we performed comparative transcriptome analysis of three rodent models of light-induced retinopathy, and we identified 37 genes that are dysregulated in all three models. Gene ontology analysis revealed that this gene set is significantly associated with a cytokine signaling axis composed of signal transducer and activator of transcription 1 and 3 (STAT1/3), interleukin 6 signal transducer (IL6ST), and oncostatin M receptor (OSMR). Furthermore, the analysis suggested that the histone acetyltransferase EP300 may be a key upstream regulator of the STAT1/3–IL6ST/OSMR axis. To examine the role of EP300 directly, we developed a larval zebrafish model of light-induced retinopathy. Using this model, we demonstrated that pharmacological inhibition of EP300 significantly increased retinal cell apoptosis, decreased photoreceptor cell outer segments, and increased proliferation of putative Müller cells upon exposure to intense light. These results suggest that EP300 may protect photoreceptor cells from light-induced damage and that activation of EP300 may be a novel therapeutic approach for the treatment of retinal degenerative diseases.

## Introduction

Retinal photoreceptor cells are uniquely adapted to function over a wide range of ambient light conditions. However, in most species, prolonged exposure to intense visible light can lead to photoreceptor cell damage (reviewed in Wenzel et al., 2005; Organisciak and Vaughan, 2010; Chen et al., 2016). Light-induced retinal damage serves as a model to study human retinal degeneration arising from environmental insult, aging, and genetic disease. Accordingly, animal models of light-induced retinopathy have been used successfully to develop therapeutic approaches for retinal degenerative diseases (Tsuruma et al., 2014; Shimazawa et al., 2015). While many of the light-induced retinopathy models share similar mechanisms, there are also significant differences (reviewed in Organisciak and Vaughan, 2010). For example, inhibition of the transcription factor (TF) activator protein-1 protects against light damage in mice (Wenzel et al., 2001) but not in T4R rhodopsin mutant dogs (Gu et al., 2009). Similarly, exposure to intense white light causes degeneration of retinal pigment epithelial cells in rats but not in mice (Remé et al., 1998). These findings suggest that there may be shared and unique pathways of light-induced retinal cell death in the various models.

Comparative transcriptome analysis has been used successfully to identify common and divergent genomic responses in a disease entity caused by various etiologies in different species (Nishimura et al., 2007, 2015b; Oka et al., 2010). Here, we sought to identify genes that are dysregulated in multiple models of light-induced retinopathy and might therefore, be exploited as novel therapeutic targets. We compared the transcriptomes of three rodent models of light-induced retinopathy (Rattner and Nathans, 2005; Natoli et al., 2010; Hadziahmetovic et al., 2012) and identified 37 genes that were differentially expressed in all three models. We also demonstrated that (i) a signaling axis composed of signal transducer and activator of transcription 1 and 3 (STAT1/3), interleukin 6 signal transducer (IL6ST), and oncostatin M receptor (STAT1/3–IL6ST/OSMR), which is involved in the neuroprotective role of IL-6 (Jung et al., 2011), plays a key role in light-induced retinopathy, (ii) the histone acetyltransferase (HAT) EP300 is a key upstream regulator of the STAT1/3–IL6ST/OSMR axis, and (iii) EP300 plays a protective role in the larval zebrafish model of light-induced retinopathy, suggesting that EP300 may be a novel therapeutic target for retinal degenerative diseases.

## Materials and methods

### Ethics statement

This study was carried out in strict accordance with Japanese law, including the Human Treatment and Management of Animals Act (2014), Standards Relating to the Care and Management of Laboratory Animals and Relief of Pain (2013), and the Guidelines for Proper Conduct of Animal Experiments (Science Council of Japan, 2006). All experiments were performed on animals under 2-phenoxyethanol anesthesia, and all efforts were made to minimize animal suffering.

### Compounds

The HAT inhibitor C646 (Bowers et al., 2010), phenylthiourea, and 5-bromo-2-deoxyuridine (BrdU) were purchased from Sigma (St. Louis, MO, USA). Stock solutions of C646 and phenylthiourea were prepared in dimethyl sulfoxide (DMSO; Nacalai Tesque, Kyoto, Japan). BrdU was dissolved in 0.3 × Danieau's solution [19.3 mM NaCl, 0.23 mM KCl, 0.13 mM MgSO_4_, 0.2 mM Ca(NO_3_)_2_, 1.7 mM HEPES, pH 7.2]. 2-Phenoxyethanol was obtained from Wako Chemicals (Osaka, Japan).

### Comparative transcriptome analysis

To identify genes that are differentially expressed in multiple rodent models of light-induced retinopathy, we analyzed three transcriptome datasets deposited in the Gene Expression Omnibus (GEO; Barrett et al., 2009), designated GSE10528 (Rattner and Nathans, 2005), GSE22818 (Natoli et al., 2010), and GSE37773 (Hadziahmetovic et al., 2012). The raw data were normalized using the packages “affy” (Gautier et al., 2004) for GSE10528 and “oligo” (Carvalho et al., 2007) for GSE22818 and GSE37773 in Bioconductor (Gentleman et al., 2004). Probes with reliable signals were selected and subjected to RankProd (Breitling et al., 2004) analysis to identify differentially expressed genes (DEGs) in each model using a false discovery rate of 10% as the threshold. The gene symbols of the DEGs were converted to the human orthologs using Life Science Knowledge Bank (World Fusion, Tokyo, Japan), and UniProt IDs were added using the ID mapping tool of UniProt (UniProtConsortium, 2015). Venn diagrams showing the number of shared and unique DEGs in the three models were drawn using PINA4MS (Cowley et al., 2012) in Cytoscape (Shannon et al., 2003).

We identified functional networks significantly related to the 37 DEGs common to the three rodent models of light-induced retinopathy by using JEPETTO (Winterhalter et al., 2014) as the integrative analytical tool, with STRING (Jensen et al., 2009) for the protein interaction network analyses and Gene Ontology (Gene Ontology Consortium, 2015), InterPro (Mitchell et al., 2015), or BioCarta (Nishimura, 2001) for the functional analyses.

To identify TFs potentially regulating the 37 common DEGs, we used iRegulon (Janky et al., 2014), which has been used successfully to identify important TFs from gene lists (Nishimura et al., 2015b; Sasagawa et al., 2016). The predicted transcriptional regulators with normalized enrichment scores (NES) >3 are shown in Table S2.

### Zebrafish

We used an albino line (Kelsh et al., 1996) obtained from the Max Planck Institute for Developmental Biology (Tübingen, Germany). Zebrafish were bred and maintained according to previously described methods (Westerfield, 2007; Nishimura et al., 2016). Briefly, zebrafish were raised at 28.5 ± 0.5°C with a 14 h/10 h light/dark cycle. Embryos were obtained and cultured in 0.3 × Danieau's solution until 6 days post-fertilization (dpf). For modeling light-induced retinopathy, zebrafish were cultured in 0.3 × Danieau's solution containing 200 μM phenylthiourea.

### A larval zebrafish model of light-induced retinopathy

Zebrafish at 3 dpf were wrapped in aluminum foil and shielded from light for 48 h. On day 5 dpf, the animals were transferred to 12-well plates (7 larvae/3 ml medium/well) and incubated under normal conditions (28°C, 14 h [7 a.m.–10 p.m.] dim light/10 h dark) or placed in a custom-made chamber (Hayashi Factory, Kyoto, Japan) to induce retinopathy (Figure S3). The chamber was 47 × 47 × 27 cm in width, length, and height, respectively, and was equipped with six fluorescent lamps (FL10ECW, Panasonic, Osaka, Japan) housed in the lid. Lamp specifications were 33 cm length, 520 lumen, 7200 kelvin, and emission peaks at 450, 540, and 610 nm. The inner walls of the chamber were lined with mirrors to expose the zebrafish to light from all directions. The multi-well plates were placed on a water-cooled plate within the chamber to maintain the animals at 27°C during light exposure.

### TUNEL staining

Terminal deoxynucleotidyl transferase dUTP nick end labeling (TUNEL) was performed using an ApopTag Fluorescein *In situ* Apoptosis Detection Kit (Millipore, Billerica, MA, USA) according to the manufacturer's protocol. Briefly, zebrafish were exposed for 24 h to intense light (13,000 lux) or normal light conditions (14 h/10 h light/dark cycle) in medium with or without 2 μM C646 and then fixed in 4% paraformaldehyde in phosphate-buffered saline (PBS; Nacalai Tesque) at 4°C overnight. Animals were washed with PBS containing 0.1% Tween 20 (PBST), incubated in water containing 3% H_2_O_2_ and 1% KOH at room temperature for 30 min, washed again with PBST, and incubated in 100% methanol at −30°C overnight. Zebrafish were rehydrated, treated with proteinase K (40 μg/ml) at 37°C for 1 h, washed with PBST, incubated in equilibration buffer at 37°C for 1 h, and then incubated in working solution containing TdT enzyme and digoxigenin-labeled dNTP at 37°C for 1 h. The animals were then washed and treated with fluorescein-labeled anti-digoxigenin IgG at 4°C overnight. Finally, zebrafish were washed once more with PBST and imaged with a SMZ25 stereomicroscope (Nikon, Tokyo, Japan) equipped with a GFP-BP filter. Quantitative analysis of the fluorescent images was performed using Volocity software (PerkinElmer, Waltham, MA, USA). The threshold fluorescence intensity for defining apoptotic areas of the retina was set at five standard deviations above the mean fluorescence intensity of the whole field of view.

### Whole-mount fluorescent immunohistochemistry

For the assessment of photoreceptor cells, zebrafish were exposed to intense or normal light conditions with or without 2 μM C646 as described above, and then fixed in 4% paraformaldehyde in PBS at 4°C overnight. Zebrafish were then washed with PBST, incubated in water containing 3% H_2_O_2_ and 1% KOH at room temperature for 30 min, washed again with PBST, and incubated in 100% methanol at −30°C overnight. After rehydration, zebrafish were treated with proteinase K (40 μg/ml) at 28°C for 30 min, washed with PBST, and then incubated in Blocking One Histo (Nacalai Tesque) at 4°C overnight. Animals were washed again with PBST and incubated in Can Get Signal Immunostain B solution (Toyobo, Osaka, Japan) containing anti-Zpr3 antibody (1:50, ZIRC, Eugene, OR, USA) at 4°C overnight. Zebrafish were washed with PBST and incubated in Can Get Signal Immunostain B solution (Toyobo) containing Alexa Fluor 488-conjugated anti-mouse IgG (1:500, Invitrogen, Carlsbad, CA) at 4°C overnight. After a final wash with PBST, animals were imaged with a SMZ25 stereomicroscope equipped with a GFP-BP filter. Quantitative analysis of the fluorescent images was performed using Volocity software. The threshold fluorescence intensity for identifying photoreceptor cell outer segments was set at four standard deviations above the mean fluorescence intensity of the whole field of view.

Retinal cell proliferation was measured as described above for the assessment of retinal cells, with the following modifications. Zebrafish were exposed to intense or normal light conditions in 0.3 × Danieau's solution containing 10 mM BrdU with or without 2 μM C646, and then fixed, dehydrated, and rehydrated as described above. After rehydration, animals were treated with proteinase K (40 μg/ml) at 37°C for 60 min, washed with PBST, and then incubated in Blocking One Histo (Nacalai Tesque) at 4°C overnight. Animals were washed again with PBST and incubated in Can Get Signal Immunostain B solution (Toyobo) containing anti-BrdU antibody (1:100, Sigma) at 4°C overnight. Zebrafish were then processed as described above except the Alexa Fluor 488-conjugated anti-mouse IgG was used at 1:200 dilution. Finally, the animals were imaged with a SMZ25 stereomicroscope equipped with a GFP-BP filter. Quantitative analysis of the fluorescent images was performed using Volocity software. The threshold fluorescence intensity for BrdU was set at three standard deviations above the mean fluorescence intensity of the whole field of view.

### Statistical analysis

Statistical analysis was performed using Prism 6 (GraphPad, La Jolla, CA, USA). For the assessment of apoptosis (TUNEL staining) and photoreceptor cell outer segments, group means were compared by analysis of variance followed by Tukey's multiple comparisons test. For the assessment of BrdU-positive cells, group means were compared by the Kruskal–Wallis test followed by Dunn's multiple comparisons test. Data are shown as the mean ± standard error (SEM).

## Results

### Identification of DEGs common to the three rodent models of light-induced retinopathy

**Figure 1:**
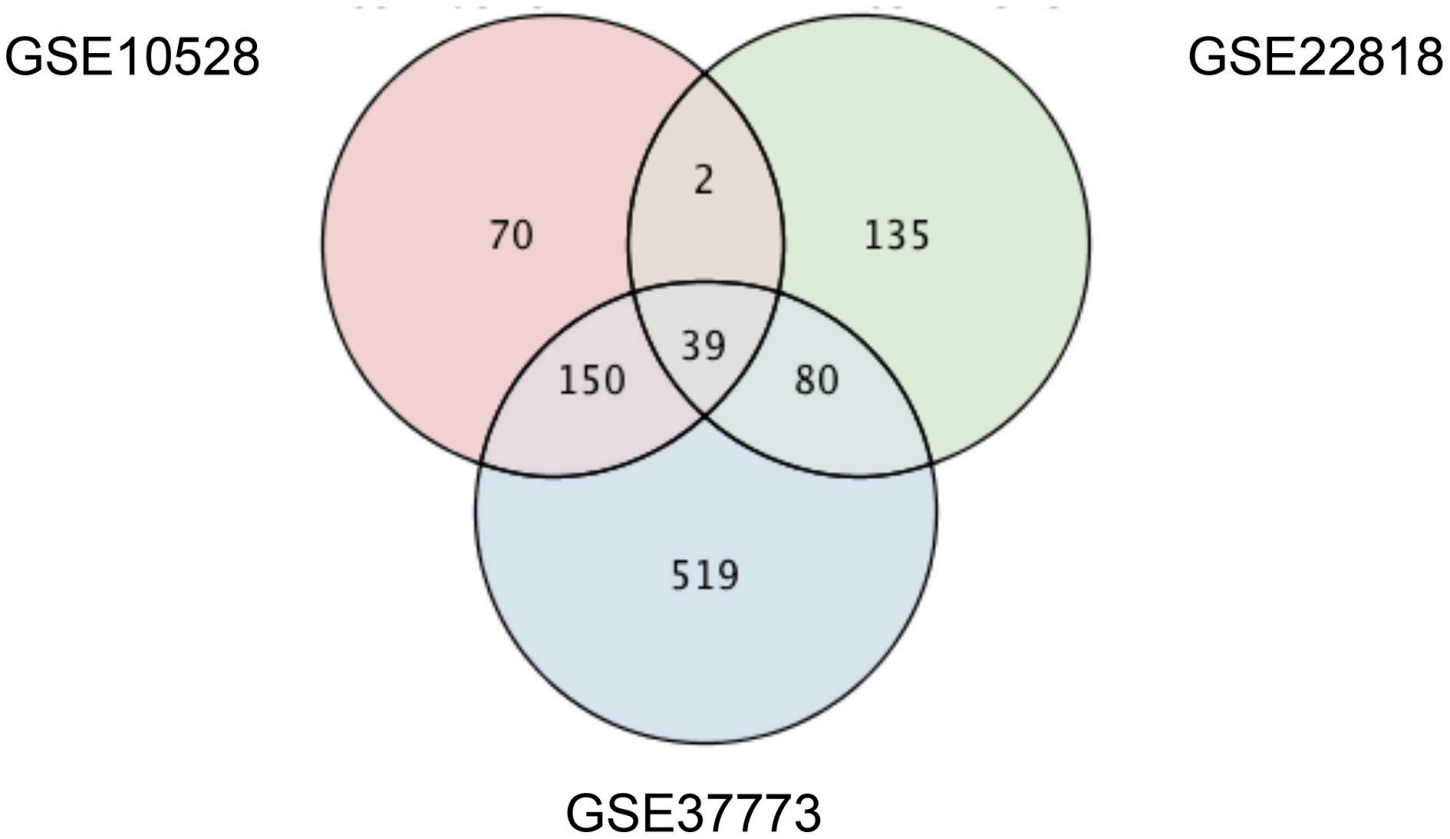
**Venn diagram of differentially expressed genes in the three rodent models of light-induced retinopathy**. Transcriptome datasets from three different rodent models of light-induced retinopathy (GSE10528, GSE22818, and GSE37773) were downloaded from a public database (GEO). Genes that were differentially expressed in the light-induced retinopathy vs. control groups were identified using a false discovery rate of 10% as the threshold. The number of differentially expressed genes in each transcriptome dataset and the overlap between datasets are shown.

To identify genes that are differentially expressed in multiple light-induced retinopathy models, we downloaded three transcriptome datasets from studies examining the retinas of rodents exposed to different light conditions. GSE10528 was from albino BALB/c mice 24 h after exposure to 6000 lux for 6 h (Rattner and Nathans, 2005), GSE22818 was from albino Sprague Dawley rats after exposure to 1000 lux for 24 h (Natoli et al., 2010), and GSE37773 was from albino BALB/c mice 4 h after exposure to 10,000 lux for 18 h (Hadziahmetovic et al., 2012). Using a false discovery rate of 10% as the threshold, we identified 261, 256, and 788 genes in GSE10528, GSE22818, and GSE37773, respectively, that were differentially expressed in animals exposed to intense light compared with normal light conditions (Table S1). Among the 39 DEGs that overlapped in the three light-induced retinopathy models (Figure 1), the change in expression was the same for 37 DEGs (Table 1), suggesting that they may be related to the pathophysiology of light-induced retinopathy.

**Table 1:** **DEGs common to the three rodent models of light-induced retinopathy**.

**Symbol**	**Name**	**GSE10528**		**GSE22818**		**GSE37773**	
		**log(light/cnt)**	**FDR**	**log(light/cnt)**	**FDR**	**log(light/cnt)**	**FDR**
A2M	Alpha-2-macroglobulin	2.39	0.00	0.83	0.06	2.70	0.00
ADAMTS1	ADAM metallopeptidase with thrombospondin type 1 motif 1	1.48	0.01	1.40	0.01	1.35	0.00
AHR	Aryl hydrocarbon receptor	1.01	0.07	1.84	0.00	1.32	0.00
AQP1	Aquaporin 1	−1.13	0.01	−1.32	0.00	−0.74	0.02
CD44	CD44 molecule	2.34	0.00	1.26	0.01	1.73	0.00
CP	Ceruloplasmin	2.14	0.00	1.25	0.02	1.95	0.00
DUSP6	Dual specificity phosphatase 6	1.69	0.00	0.96	0.04	0.73	0.02
EBPL	Emopamil binding protein-like	−0.92	0.04	−1.18	0.00	−0.96	0.00
EDN2	Endothelin 2	3.69	0.00	0.99	0.03	0.76	0.02
FOS	FBJ murine osteosarcoma viral oncogene homolog	1.14	0.08	1.28	0.01	1.00	0.00
IFITM2	Interferon induced transmembrane protein 2	1.31	0.01	1.64	0.01	2.45	0.00
IL6ST	Interleukin 6 signal transducer	1.15	0.03	1.10	0.02	1.21	0.00
IPO5	Importin 5	1.25	0.01	0.98	0.03	1.13	0.00
IRF9	Interferon regulatory factor 9	1.86	0.00	2.35	0.00	2.30	0.00
ITGB1	Integrin, beta 1	1.00	0.07	0.76	0.10	0.59	0.05
JUN	Jun proto-oncogene	1.57	0.00	2.04	0.00	1.42	0.00
LGALS3	Lectin, galactoside-binding, soluble, 3	3.14	0.00	1.30	0.02	0.80	0.01
LITAF	Lipopolysaccharide-induced TNF factor	0.97	0.08	1.91	0.00	0.68	0.03
MSN	Moesin	2.19	0.00	1.03	0.04	1.97	0.00
NRL	Neural retina leucine zipper	−1.04	0.02	−1.12	0.01	−0.81	0.01
OPTN	Optineurin	−0.91	0.04	−0.96	0.02	−0.69	0.03
OSMR	Oncostatin M receptor	3.04	0.00	2.59	0.00	2.61	0.00
PDPN	Podoplanin	2.78	0.00	1.99	0.00	1.79	0.00
PITPNM3	PITPNM family member 3	−1.37	0.00	−0.84	0.08	−0.71	0.02
PROS1	Protein S (alpha)	0.96	0.09	1.06	0.02	1.72	0.00
RDH10	retinol dehydrogenase 10 (all-trans)	1.00	0.07	1.58	0.00	0.50	0.10
REEP6	Receptor accessory protein 6	−1.23	0.01	−1.70	0.00	−1.30	0.00
S100A10	S100 calcium binding protein A10	2.44	0.00	0.91	0.06	2.03	0.00
S100A6	S100 calcium binding protein A6	2.45	0.00	1.16	0.03	3.62	0.00
SLC14A1	Solute carrier family 14, member 1	2.17	0.00	1.41	0.01	2.57	0.00
SLC24A1	Solute carrier family 24 member 1	−0.85	0.06	−0.98	0.03	−0.65	0.04
STAT1	Signal transducer and activator of transcription 1, 91kDa	2.00	0.00	1.60	0.01	2.17	0.00
STAT3	Signal transducer and activator of transcription 3	1.92	0.00	2.03	0.00	1.96	0.00
SUSD3	Sushi domain containing 3	−1.06	0.02	−1.18	0.00	−0.74	0.02
TAGLN2	Transgelin 2	0.98	0.09	0.96	0.05	1.03	0.00
TARS	Threonyl-tRNA synthetase	1.06	0.05	1.22	0.01	0.66	0.03
TRIM25	Tripartite motif containing 25	1.01	0.07	0.97	0.04	1.53	0.00

*The changes of gene expression increased and decreased in light-induced retinopathy are shown in red and blue, respectively*.

### Identification of STAT1/3–IL6ST/OSMR as a key signaling pathway in light-induced retinopathy

**Figure 2:**
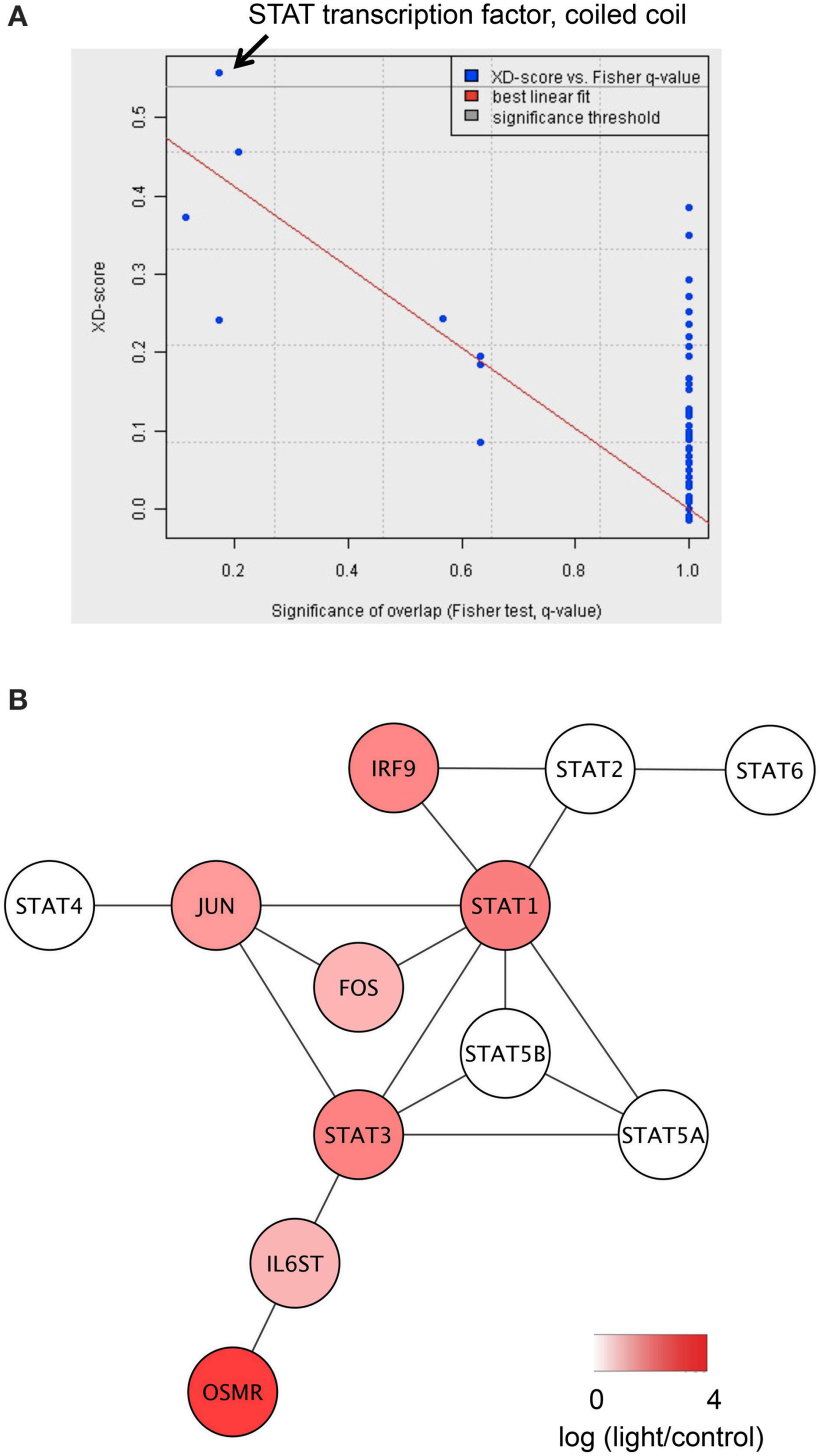
**Identification of “STAT transcription factor, coiled coil” as the domain most significantly associated with the DEGs common to the three rodent models of light-induced retinopathy. (A)** Scatter plot of domains in InterPro based on the network-based association score (XD score) and the significance of overlap (*q*-value) using the 37 common DEGs as the input in JEPETTO. The most significant domain was “STAT transcription factor, coiled coil.” **(B)** The “STAT transcription factor, coiled coil” network identified by JEPETTO. The seven genes with increased expression in the light-induced retinopathy models are shown in red.

We next investigated which functional networks were significantly associated with the 37 common DEGs using the JEPETTO analytical plugin for Cytoscape (Winterhalter et al., 2014). JEPETTO performs integrated analyses using protein interaction networks such as STRING (Jensen et al., 2009) and various databases such as Gene Ontology (Gene Ontology Consortium, 2015), InterPro (Mitchell et al., 2015), and BioCarta (Nishimura, 2001) to find functional networks significantly related to a given gene list. Using JEPETTO with InterPro, we found that “STAT transcription factor, coiled coil” was significantly associated with the 37 common DEGs (XD score 0.56 and *q*-value 1.7 × 10^−1^, Figure 2). Among the 12 genes included in the “STAT transcription factor, coiled coil” network, 7 genes (*STAT1, STAT3, IL6ST, OSMR, JUN, FOS*, and *IRF9*) were significantly upregulated in the three rodent models of light-induced retinopathy. STAT1 and STAT3 can form homodimers or heterodimers (reviewed in Kisseleva et al., 2002). A coiled coil domain important for protein–protein interactions is conserved in all members of the STAT family (reviewed in Reich, 2013). Interleukin 6 signal transducer (IL6ST), also known as GP130, can form heterodimers with the oncostatin M receptor (OSMR; Hermanns et al., 1999). Activation of STAT3 increases transcription of IL6ST (O'Brien and Manolagas, 1997) and OSMR (Traber et al., 2015) and, in turn, activation of IL6ST/OSMR leads to STAT1/3 activation (Dreuw et al., 2004). These findings suggest that STAT1/3 and IL6ST/OSMR may form a positive feedback loop. Using JEPETTO in combination with Gene Ontology and BioCarta, we found that “IL 6 signaling pathway” (XD score 0.70 and *q*-value 5.8 × 10^−4^, Figure S1) and “growth factor binding” (XD score 0.50 and *q*-value 2.6 × 10^−2^, Figure S2), respectively, were significantly associated with the 37 common DEGs. *STAT1, STAT3, IL6ST, OSMR, JUN, FOS*, and *IRF9* are represented in the “STAT transcription factor, coiled coil,” “IL 6 signaling pathway,” and “growth factor binding” networks. These results suggest that STAT1/3–IL6ST/OSMR may be a key signaling pathway in the pathophysiology of light-induced retinopathy.

### Identification of EP300 as a key transcription factor in light-induced retinopathy

**Figure 3:**
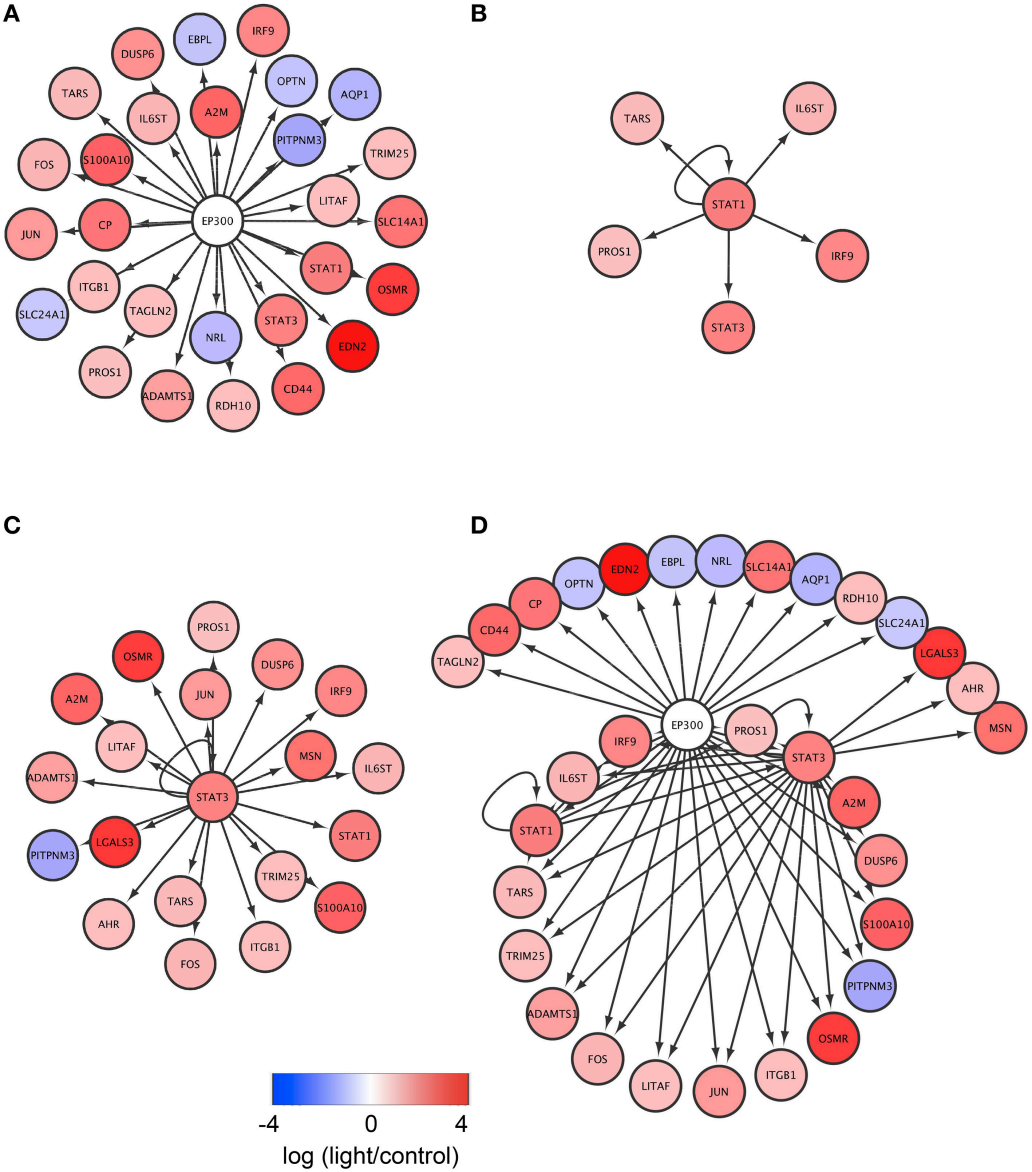
**Identification of EP300, STAT1, and STAT3 as the key transcription factors potentially regulating the DEGs common to the three rodent models of light-induced retinopathy. (A–C)** The DEGs common to the three models and potentially regulated by EP300 **(A)**, STAT1 **(B)**, and STAT3 **(C)**. **(D)** The union of the EP300, STAT1, and STAT3 networks and their potential targets.

We hypothesized that the 37 DEGs common to the three rodent models of light-induced retinopathy might be regulated by specific TFs. To test this, we analyzed the gene sets using iRegulon, which exploits the fact that genes co-regulated by the same TF contain common TF binding sites and uses gene sets derived from ENCODE ChIP-seq data (Gerstein et al., 2012; Janky et al., 2014). Using iRegulon, we identified six TFs with significant NES (>3), suggesting that they regulate the expression of common DEGs (Table S2). EP300, STAT1, and STAT3 were the first, second, and third ranked TFs by significance. iRegulon identified 28 genes (Figure 3A), 6 genes (Figure 3B), and 20 genes (Figure 3C) as potential transcriptional targets of EP300, STAT1, and STAT3, respectively. The union of the EP300, STAT1, and STAT3 networks and their potential transcriptional targets is shown in Figure 3D. Of the potential targets, 17 genes, including *STAT1, STAT3, IL6ST*, and *OSMR*, were targets of EP300 and either STAT1 or STAT3. These results suggest that EP300 and STAT1/3 may regulate the expression of the 16 genes cooperatively and that EP300 may be a key TF in light-induced retinopathy.

### Inhibition of EP300 increases apoptosis in the zebrafish model of light-induced retinopathy

**Figure 4:**
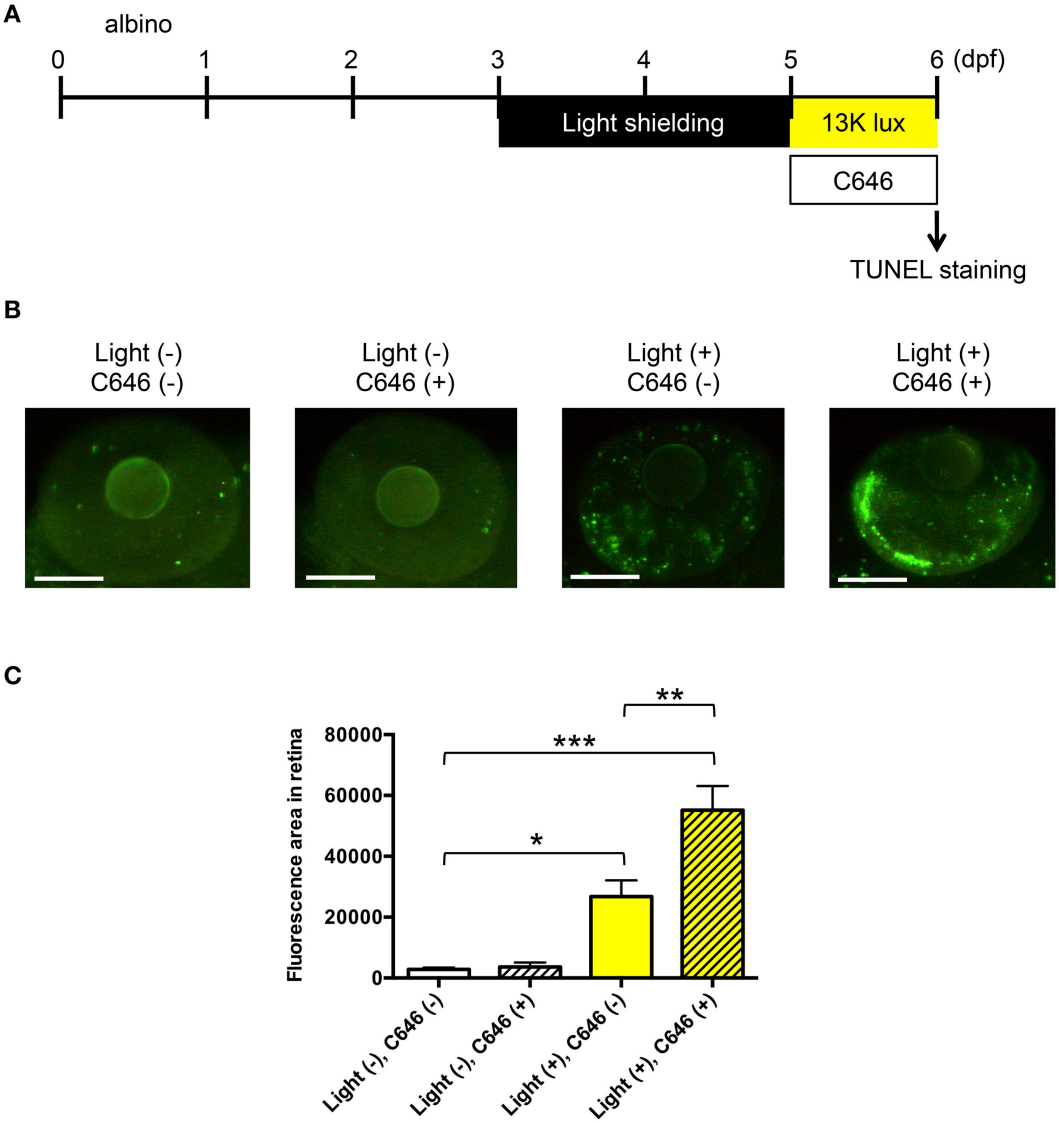
**Inhibition of EP300 increases retinal apoptosis in a larval zebrafish model of light-induced retinopathy**. **(A)** Protocol for light-induced retinal damage in larval zebrafish. Zebrafish are shielded from light between 3 and 5 days post-fertilization (dpf) and then exposed to normal conditions (14 h 250 lux/10 h dark) or intense light (13,000 lux) in the presence or absence of 2 μM C646 for 24 h at 27°C. After light exposure, whole-mount TUNEL staining was performed. **(B)** Representative images of TUNEL staining in the retina of zebrafish exposed to intense light (indicated as light+) or normal light conditions (light-). Scale bars, 100 μm. **(C)** Quantitative analysis of retinal apoptosis in zebrafish exposed to the conditions shown in **(B)**. ^*^p < 0.01, ^**^p < 0.001, ^***^p < 0.0001. Data are the mean ± SEM of 13–14 zebrafish/group.

To examine the function of EP300 in light-induced retinopathy, we developed a novel model using larval zebrafish (Figures S3 and Figure 4A). Larval zebrafish were first shielded from light for 48 h between 3 and 5 dpf and then exposed to intense light (13,000 lux for 24 h) or housed under normal conditions (14 h 250 lux/10 h dark) in the absence or presence of C646, a specific inhibitor of EP300 (Bowers et al., 2010). At the end of the 24 h incubation, retinal apoptosis was examined by TUNEL staining. The retinas of zebrafish exposed to intense light had significantly higher apoptosis levels than the retinas of zebrafish housed under normal conditions (Figures 4B,C). Moreover, C646 treatment further increased the level of apoptosis in the retinas of zebrafish exposed to intense light, whereas it had no effect on zebrafish housed under normal light conditions (Figure 4C). To exclude the possibility that C646 might induce non-specific cell death following light exposure, we measured apoptosis in the forebrain. However, we found no significant difference in the apoptotic signals between the forebrains of zebrafish exposed to intense light with and without C646 treatment (data not shown). These results suggest that activated EP300 may protect the retina by reducing apoptosis during intense light exposure.

### Inhibition of EP300 increases loss of photoreceptor cell outer segments in the zebrafish model of light-induced retinopathy

**Figure 5:**
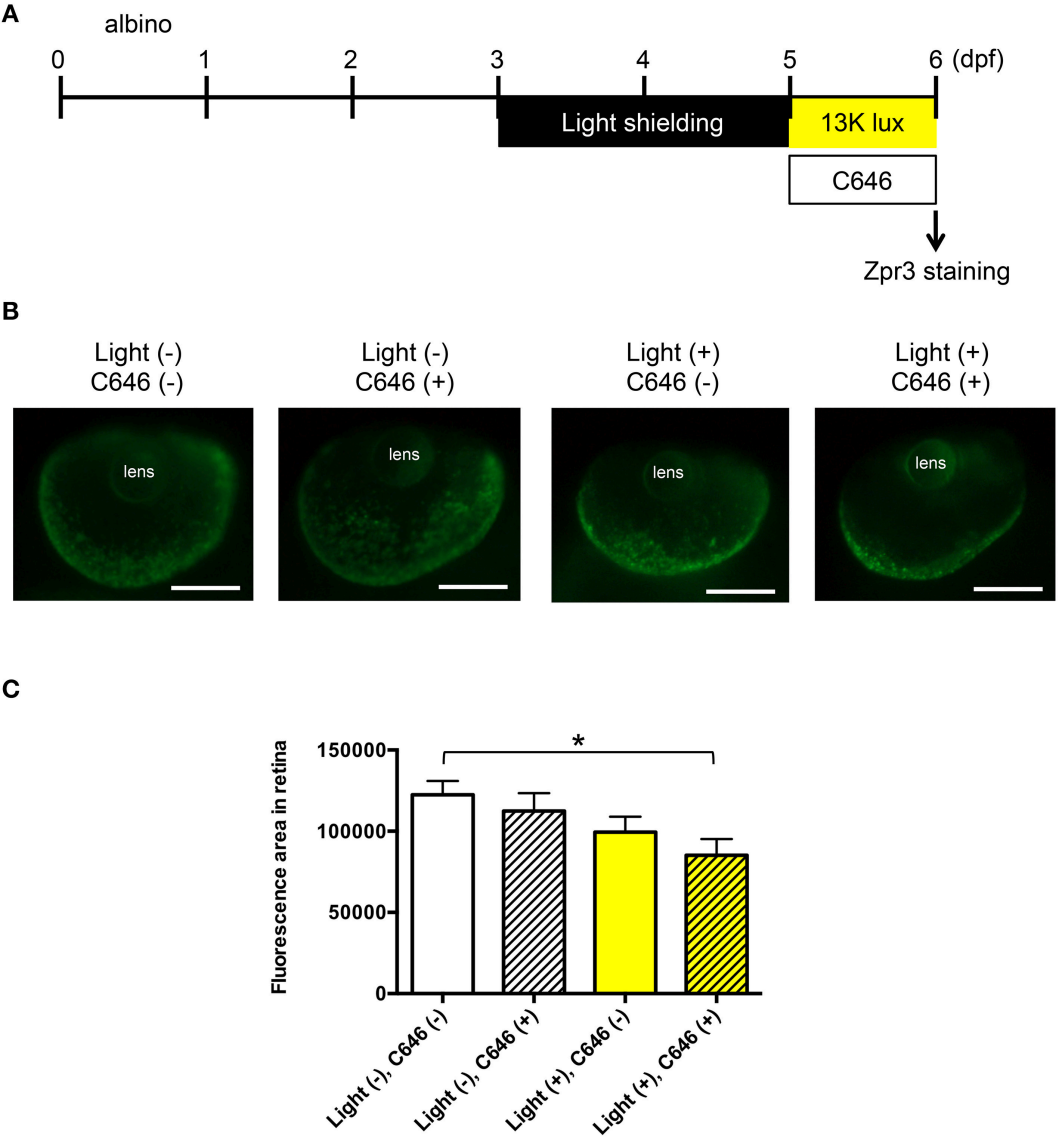
**Inhibition of EP300 reduces the photoreceptor cell outer segments in a zebrafish model of light-induced retinopathy. (A)** Protocol for light-induced retinal damage in larval zebrafish, as described for Figure 4A. After light exposure, whole-mount immunohistochemical staining with anti-Zpr3 antibody was performed. **(B)** Representative images of anti-Zpr3 antibody staining of zebrafish exposed to normal or intense light. Scale bars, 100 μm. **(C)** Quantitative analysis of photoreceptor cell outer segments of zebrafish exposed to the conditions shown in **(B)**. ^*^p < 0.05. Data are the mean ± SEM of *n* = 24 zebrafish/group for light−/C646- and light−/C646+, *n* = 17 for light+/C646−, and *n* = 22 for light+/C646+.

To further investigate the role of EP300 in retinal protection, we performed whole-mount immunohistochemical staining of larval zebrafish with light-induced retinopathy (Figure 5A) using anti-Zpr3 antibody, which detects both rod and cone photoreceptor cell outer segments (Yin et al., 2012). As shown in Figure 5B, the fluorescent signal from the photoreceptor cell outer segments, especially in the area between the rim and the lens, was decreased in zebrafish exposed to intense light in the presence of C646. Quantitative analysis of the fluorescent signal revealed a significant reduction in the area of photoreceptor cell outer segments in zebrafish exposed to intense light in the presence of C646 compared with zebrafish housed under normal conditions (Figure 5C). The retina diameters were not significantly different among the four groups (data not shown). These results support a role for EP300 in protecting the retina from light-induced damage, at least in zebrafish.

### Inhibition of EP300 increases BrdU incorporation in putative müller cells in the zebrafish model of light-induced retinopathy

**Figure 6:**
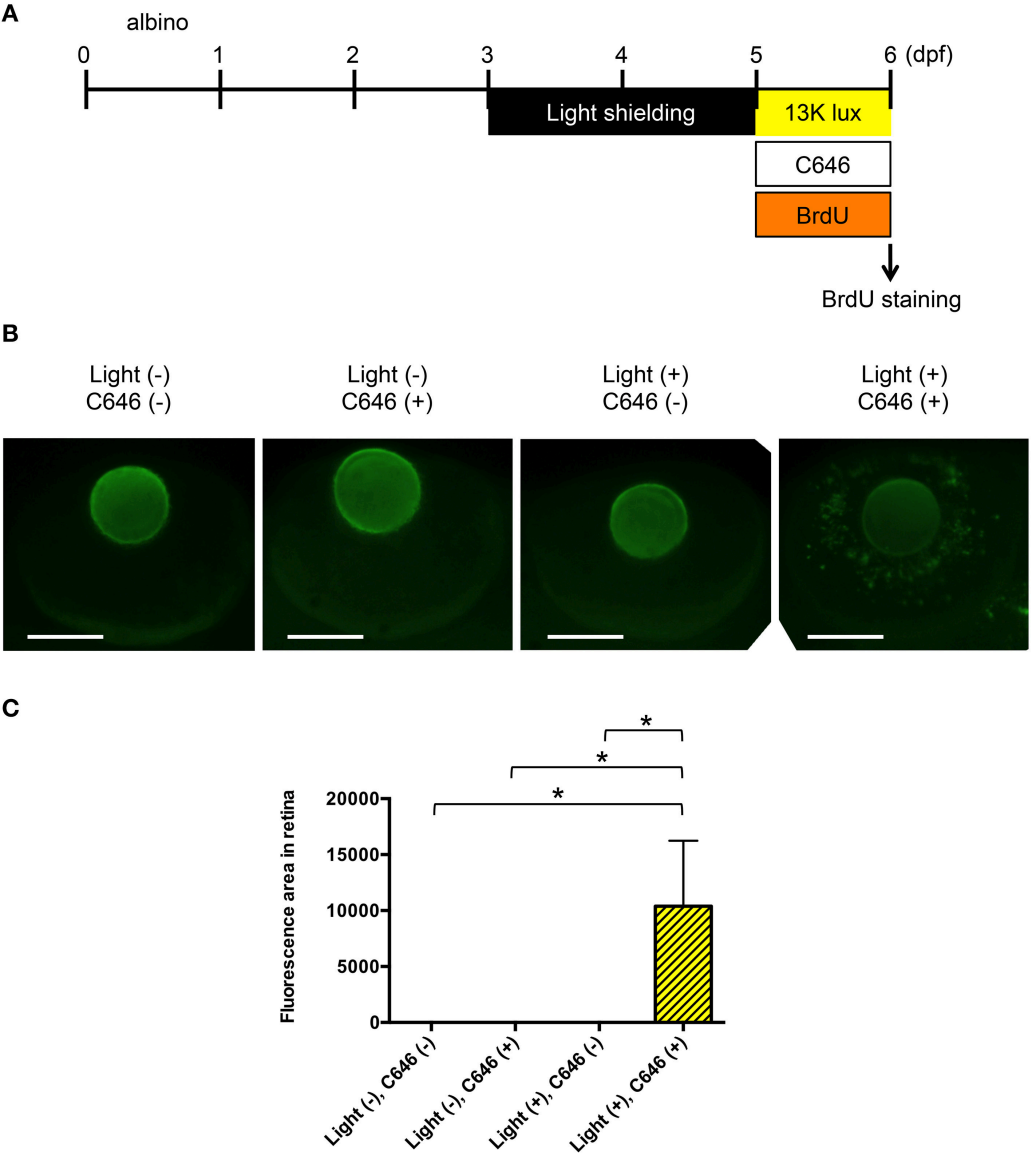
**Inhibition of EP300 increases BrdU incorporation in putative Müller cells in the zebrafish model of light-induced retinopathy. (A)** Protocol for light-induced retinal damage in larval zebrafish, as described for Figure 4A. After light exposure, whole-mount immunohistochemical staining with anti-BrdU antibody was performed. **(B)** Representative images of anti-BrdU antibody staining of zebrafish exposed to normal or intense light. Scale bars, 100 μm. **(C)** Quantitative analysis of BrdU-positive cells in the retinas of zebrafish exposed to the conditions shown in **(B)**. ^*^p < 0.05. Data are the mean ± SEM of *n* = 8 for all groups.

Following retinal injury, zebrafish Müller glia proliferate and differentiate into the retinal cell types lost through injury (Lenkowski and Raymond, 2014). To determine whether Müller cells proliferate in zebrafish with light-induced retinopathy, zebrafish were immersed in medium containing BrdU with or without C646 and/or exposed to intense light for 1 day (Figure 6A), after which, whole-mount immunohistochemical staining with anti-BrdU antibody was performed. As shown in Figures 6B,C, there was a significant increase in the appearance of BrdU-positive cells with an elongated morphology, consistent with the morphology of proliferating Müller cells (Yurco and Cameron, 2005), in zebrafish treated with C646 and exposed to intense light. These results suggest that inhibition of EP300 may increase retinal injury in the zebrafish model of light-induced retinopathy, thereby stimulating the proliferation of Müller glia.

## Discussion

The results of this study suggest that (i) the STAT1/3–IL6ST/OSMR axis is a key signaling pathway in three rodent models of light-induced retinopathy, (ii) EP300 is a key upstream regulator of the STAT1/3–IL6ST/OSMR axis, and (iii) EP300 plays a protective role in a larval zebrafish model of light-induced retinopathy.

### The STAT1/3–IL6ST/OSMR axis is a key signaling pathway in light-induced retinopathy

We identified 37 genes differentially regulated in the three rodent models of light-induced retinopathy, and found significant enrichment of these genes in “STAT transcription factor, coiled coil,” “IL 6 signaling pathway,” and “growth factor binding” networks. *STAT1, STAT3, IL6ST*, and *OSMR* were represented in all three networks and the expression of these genes was increased in the three rodent models studied here.

Previous work has demonstrated that the expression of STAT1 and STAT3 is increased in light-induced retinopathy (Samardzija et al., 2006) and that the increase in STAT3 is not observed in leukemia inhibitory factor (LIF) null mice (Bürgi et al., 2009). In the retina of BALB/c mice, expression of LIF, which is a member of the IL-6 family (Heinrich et al., 2003), is markedly increased by light exposure, peaking at 6 to 12 h after exposure (Samardzija et al., 2006). These findings suggest that the increased expression of STAT1 and STAT3 observed in the three light-induced retinopathy models examined here may be mediated by LIF. STAT1 and STAT3 can bind to the promoter of *IL6ST* as homodimers or heterodimers and positively regulate its transcription (O'Brien and Manolagas, 1997). Activation of STAT3 also increases transcription of *OSMR* (Traber et al., 2015). Therefore, the retinopathy-associated increase in *IL6ST* and *OSMR* detected here may be a result of STAT1/3-mediated transcriptional activation.

IL6ST and OSMR have also been shown to form heterodimers (Hermanns et al., 1999), and activation of such heterodimers leads to STAT1/3 activation (Dreuw et al., 2004). Given that *STAT1, STAT3, IL6ST*, and *OSMR* are all differentially upregulated in the models studied here, these reports are consistent with the hypothesis that the STAT1/3–IL6ST/OSMR axis is activated in light-induced retinopathy. Knockout of IL6ST increases photoreceptor cell death not only in a light-induced retinal damage model (Ueki et al., 2009) but also in other photoreceptor degeneration models such as retinitis pigmentosa and age-related macular degeneration (Rhee et al., 2013). Photoreceptor-specific deletion of STAT3 accelerates photoreceptor degeneration, whereas the expression of dominant active STAT3 improves photoreceptor survival in two models of retinitis pigmentosa (Jiang et al., 2014). Collectively, these findings are consistent with the possibility that STAT1/3–IL6ST/OSMR axis may be activated in light-induced retinopathy as an endogenous anti-apoptotic signaling pathway.

### EP300 is a key upstream regulator of the STAT1/3–IL6ST/OSMR axis

Our study identified 16 genes, including *STAT1, STAT3, IL6ST*, and *OSMR*, as potential transcriptional targets of EP300 and either STAT1 or STAT3, suggesting that EP300 and STAT1/3 may act cooperatively to regulate expression of the 16 genes in light-induced retinopathy.

EP300 is a transcriptional cofactor with intrinsic acetyltransferase activity (reviewed in Ghosh and Varga, 2007). The activity of STAT1 and STAT3 is known to be regulated by EP300-dependent acetylation (Yuan et al., 2005; Hou et al., 2008; Kotla and Rao, 2015; reviewed in Zhuang, 2013), and complexes of EP300 and STAT3 can bind to the promoters of *IL6ST* (Chen et al., 2008) and glial fibrillary acidic protein (*GFAP*) (Nakashima et al., 1999) and regulate their expression. In addition, inhibition of EP300 by C646 reduces phosphorylation of STAT3 at tyrosine 705 by angiotensin II (Ni et al., 2014). Tyrosine 705 phosphorylation induces STAT3 translocation to the nucleus and activates its transcriptional activity (Wen et al., 1995). Taken together with these reports, our findings support a role for EP300 as a key modulator of the anti-apoptotic effects of the endogenously activated STAT1/3–IL6ST/OSMR axis in light-induced retinopathy.

Acetylation of STAT3 can be regulated not only by HATs such as EP300 and CREB binding protein but also by HDACs (reviewed in Choi and Reddy, 2011; Zhuang, 2013). Sirtuin 1, a member of the sirtuin family of HDACs, deacetylates STAT3, resulting in decreased STAT3 tyrosine phosphorylation and transcriptional activation (Nie et al., 2009). Excessive HDAC activation plays a critical role in neurodegeneration in a mouse model of retinitis pigmentosa (Sancho-Pelluz et al., 2010), and several HDAC inhibitors have neuroprotective effects in retinal degenerative diseases (reviewed in Zhang et al., 2015). Thus, activation of HATs and/or inhibition of HDACs to increase STAT3 acetylation may be a potential therapeutic approach for retinal degenerative diseases resembling light-induced retinopathy.

### EP300 is protective in a larval zebrafish model of light-induced retinopathy

In this study, we demonstrated that inhibition of EP300 by C646 significantly increased (i) retinal apoptosis, (ii) photoreceptor damage, and (iii) proliferation of putative Müller cells in a larval zebrafish model of light-induced retinopathy.

Inhibition of photoreceptor apoptosis is an important strategy to protect the morphology and function of the retina in retinal degenerative diseases (Samardzija et al., 2006). Application of ciliary neurotrophic factor (CNTF) and fibroblast growth factor 2 (FGF2) to the retina significantly reduces apoptosis (Kassen et al., 2009; Kolomeyer and Zarbin, 2014) and increases the survival of photoreceptor cells in light-induced retinopathy (reviewed in Organisciak and Vaughan, 2010). Moreover, the interaction between EP300 and STAT3 is increased by treatment with CNTF (Hong and Song, 2014) or FGF2 (Cheng et al., 2005), suggesting a possible mechanism whereby CNTF and FGF2 reduce apoptosis and protect photoreceptor cells from light-induced retinopathy. Further studies are required to find small molecules that can activate EP300 and to examine their effects on apoptosis in light-induced retinopathy.

In this study, we described a new larval zebrafish model of light-induced retinopathy. Although similar models have been successfully implemented with adult zebrafish (reviewed in Gorsuch and Hyde, 2014), we are aware of only one other report demonstrating that larval zebrafish can be used to model light-induced retinopathy (Meyers et al., 2012). In that study, larval zebrafish in a 50 ml beaker were exposed to light using a liquid fiber optic light line connected to a metal-halide microscope illuminator. In our system, four multi-well plates can be illuminated at the same time, allowing chemical screening to be performed in a relatively high-throughput manner.

The zebrafish and human retinas are very similar (reviewed in Chhetri et al., 2014; Nishimura et al., 2015a). The neuronal cell bodies in both zebrafish and humans are precisely organized in three major laminae that are separated by plexiform layers. Similar to humans, zebrafish are diurnal. Zebrafish also have blood–brain and blood–retinal barriers (Watanabe et al., 2012; Nishimura et al., 2013). There are a few differences, however, between the visual systems of zebrafish and humans; for example, zebrafish have lateral eyes with reduced binocular overlap in the visual fields, less densely populated ganglion cells, absent fovea, and tetrachromatic vision (Chhetri et al., 2014). Understanding the similarities and differences between zebrafish and human vision is critical if the zebrafish model is to be useful for understanding the pathophysiology of human retinal diseases and for developing novel therapies. Many transgenic lines expressing fluorescent proteins in specific retinal subpopulations have also been developed and are available through public resources, enabling various approaches to *in vivo* imaging of the retina of larval zebrafish (Nishimura et al., 2015a). Knockout and knockin of specific genes can also be performed using transcription activator-like effector nuclease (TALEN) and clustered regularly interspaced short palindromic repeats (CRISPR)-Cas9 systems (Auer and Del Bene, 2014). When used in combination with these advanced technologies, the larval zebrafish model of light-induced retinopathy developed in this study will be a powerful tool in the search for therapeutic compounds that can protect photoreceptors from degenerative retinal diseases.

## Author contributions

YN conceived the study, performed the bioinformatics analyses, developed the larval zebrafish model of light-induced retinopathy, and wrote the manuscript. RK developed the larval zebrafish model of light-induced retinopathy and validated the effect of EP300. YA, SS, SM, MY, SO, and KK helped with the experiments. HY, KM, SY, KT, MS, and HH provided essential conceptual help in fabricating the light-induced retinopathy chamber. TT conceived the study, developed the larval zebrafish model of light-induced retinopathy, and wrote the manuscript.

## Funding

This work was supported in part by JSPS KAKENHI (25670127, 15K15051, 24510069), JST A-step (AS262Z00004Q), and the Long-range Research Initiative of the Japan Chemical Industrial Association (13_PT01-01).

### Conflict of interest statement

The authors declare that the research was conducted in the absence of any commercial or financial relationships that could be construed as a potential conflict of interest.

## Supplementary material

Table S1 **DEGs in each rodent model of light-induced retinopathy**. S1-1 DEGs in a mouse model of light-induced retinopathy (GSE10528). S1-2 DEGs in a rat model of light-induced retinopathy (GSE22818). S1-3 DEGs in a mouse model of light-induced retinopathy (GSE37773).

Table S2 **Transcription factors potentially regulating DEGs common to the three rodent models of light-induced retinopathy**.

Figure S1 **Identification of “IL 6 signaling pathway” as the pathway most significantly associated with the DEGs common to the three rodent models of light-induced retinopathy. (A)** Scatter plot of pathways in Biocarta based on the network-based association score (XD score) and the significance of overlap (*q*-value) using the 37 common DEGs as the input in JEPETTO. The most significant domain was “IL 6 signaling pathway.” **(B)** The “IL 6 signaling pathway” network identified by JEPETTO. The nine genes with increased expression in the light-induced retinopathy models are shown in red.

Figure S2 **Identification of “growth factor binding” as the molecular function most significantly associated with the DEGs common to the three rodent models of light-induced retinopathy. (A)** Scatter plot of molecular functions in Gene Ontology based on the network-based association score (XD score) and the significance of overlap (*q*-value) using the 37 common DEGs as the input in JEPETTO. The most significant domain was “growth factor binding.” **(B)** The “growth factor binding” network identified by JEPETTO. The eight genes with increased expression in the light-induced retinopathy models are shown in red.

Figure S3 **The chamber fabricated for the larval zebrafish model of light-induced retinopathy**.
