# Supplementary figures and images for: EP300 Protects from Light-Induced Retinopathy in Zebrafish
Source: Front Pharmacol. 2016 May 19;7:126. doi: 10.3389/fphar.2016.00126 (PMC4871856; doi:10.3389/fphar.2016.00126)

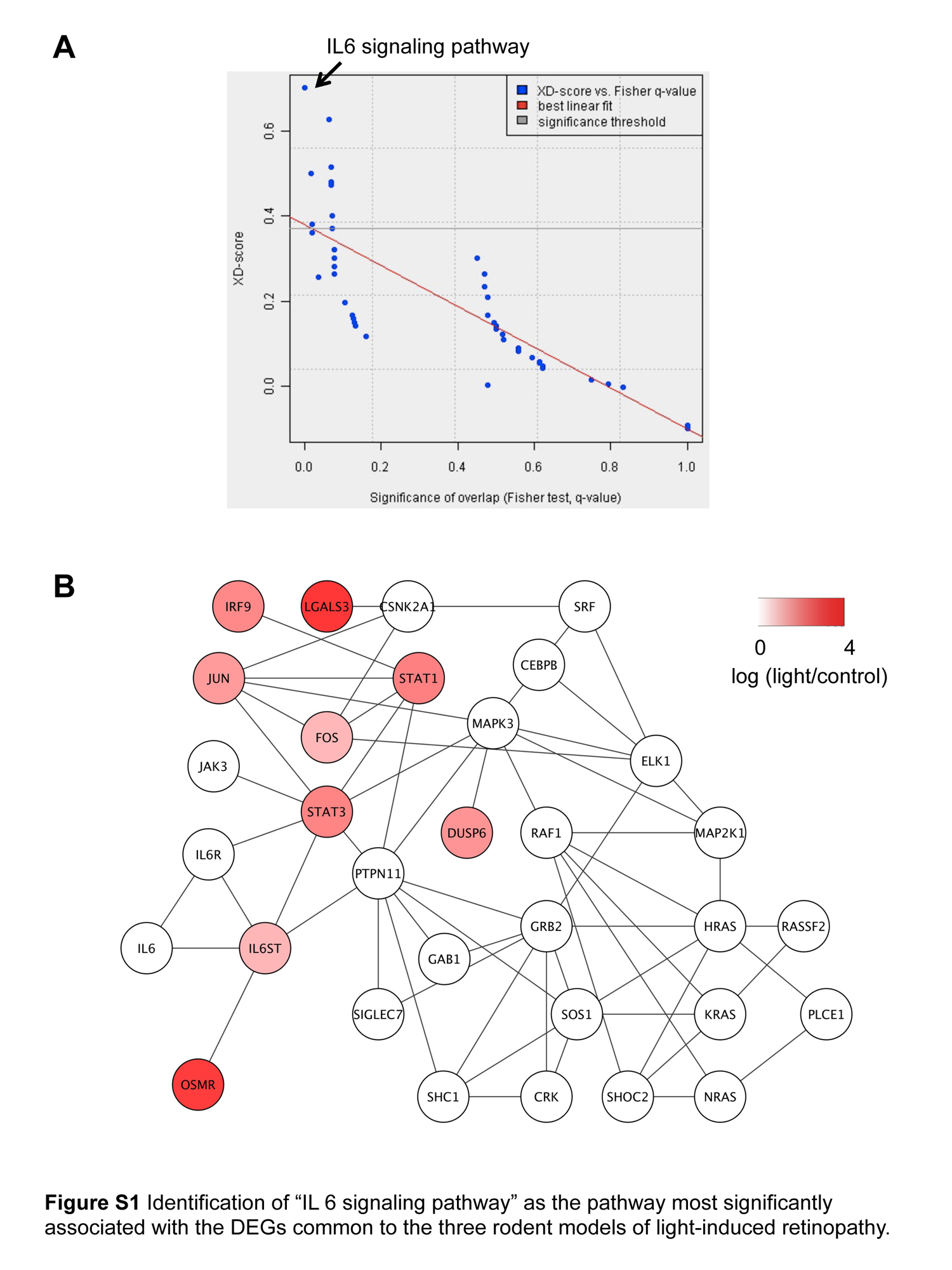

Supplement: Figure S1 — Identification of “IL 6 signaling pathway” as the pathway most significantly associated with the DEGs common to the three rodent models of light-induced retinopathy. (A) Scatter plot of pathways in Biocarta based on the network-based association score (XD score) and the significance of overlap (q-value) using the 37 common DEGs as the input in JEPETTO. The most significant domain was “IL 6 signaling pathway.” (B) The “IL 6 signaling pathway” network identified by JEPETTO. The nine genes with increased expression in the light-induced retinopathy models are shown in red. [file Image1.jpg]

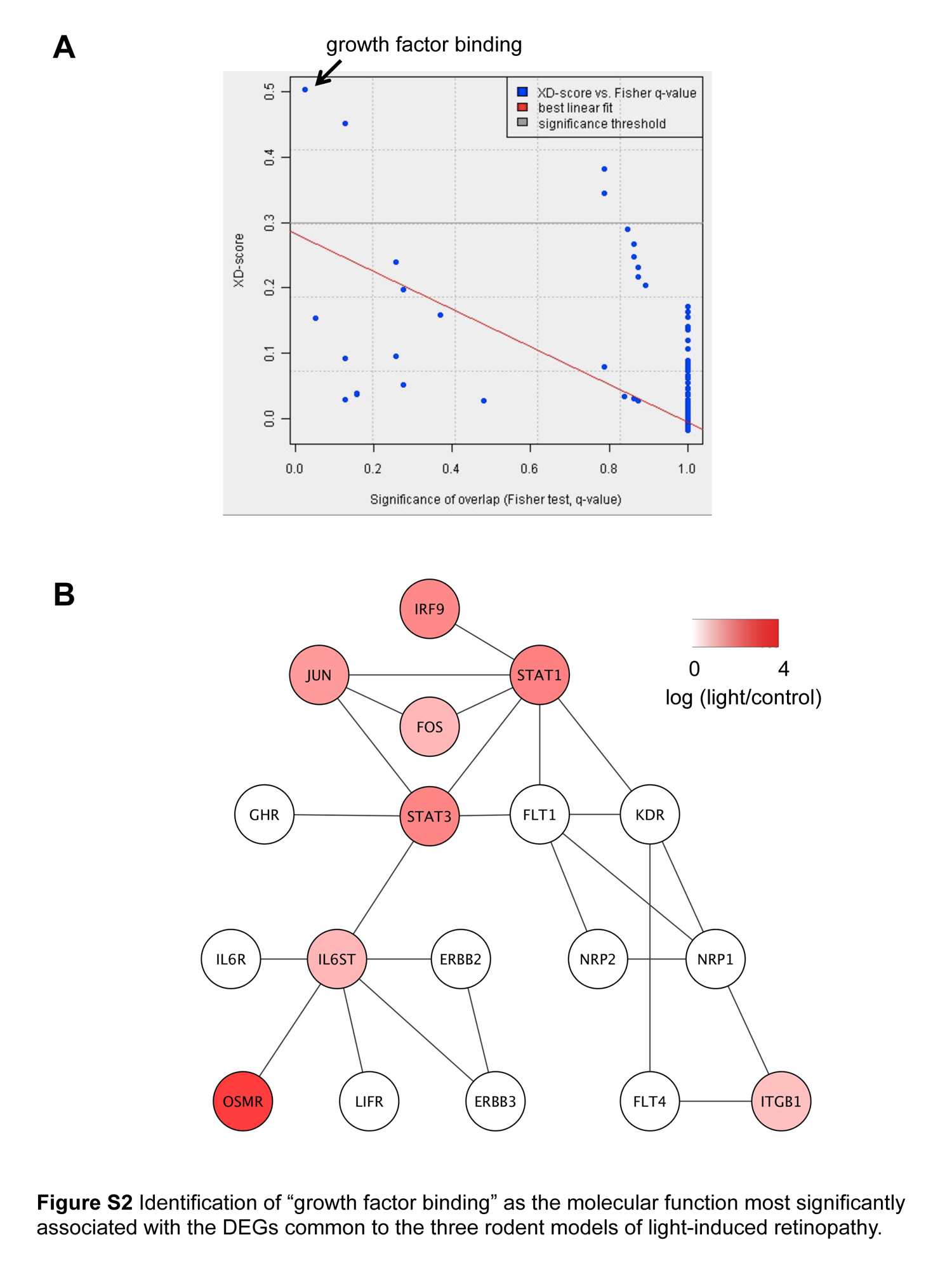

Supplement: Figure S2 — Identification of “growth factor binding” as the molecular function most significantly associated with the DEGs common to the three rodent models of light-induced retinopathy. (A) Scatter plot of molecular functions in Gene Ontology based on the network-based association score (XD score) and the significance of overlap (q-value) using the 37 common DEGs as the input in JEPETTO. The most significant domain was “growth factor binding.” (B) The “growth factor binding” network identified by JEPETTO. The eight genes with increased expression in the light-induced retinopathy models are shown in red. [file Image2.jpg]

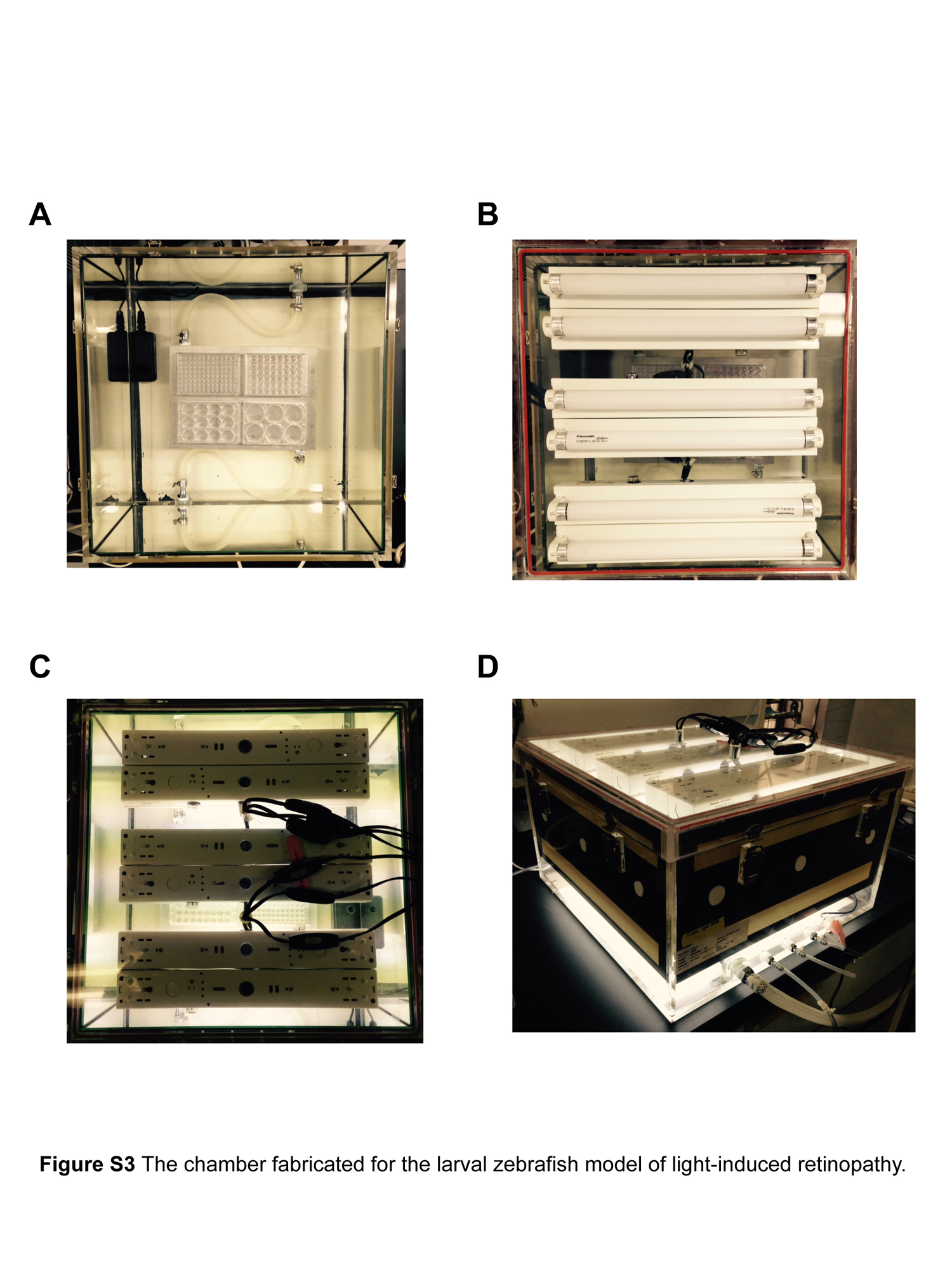

Supplement: Figure S3 — The chamber fabricated for the larval zebrafish model of light-induced retinopathy. [file Image3.jpg]
